# Equal Opportunity for Low-Degree Network Nodes: A PageRank-Based Method for Protein Target Identification in Metabolic Graphs

**DOI:** 10.1371/journal.pone.0054204

**Published:** 2013-01-29

**Authors:** Dániel Bánky, Gábor Iván, Vince Grolmusz

**Affiliations:** 1 Protein Information Technology Group, Eötvös University, Pázmány Péter stny. 1/C, Budapest, Hungary; 2 Uratim Ltd., Budapest, Hungary; Semmelweis University, Hungary

## Abstract

Biological network data, such as metabolic-, signaling- or physical interaction graphs of proteins are increasingly available in public repositories for important species. Tools for the quantitative analysis of these networks are being developed today. Protein network-based drug target identification methods usually return protein hubs with large degrees in the networks as potentially important targets. Some known, important protein targets, however, are not hubs at all, and perturbing protein hubs in these networks may have several unwanted physiological effects, due to their interaction with numerous partners. Here, we show a novel method applicable in networks with directed edges (such as metabolic networks) that compensates for the low degree (non-hub) vertices in the network, and identifies important nodes, regardless of their hub properties. Our method computes the PageRank for the nodes of the network, and divides the PageRank by the in-degree (i.e., the number of incoming edges) of the node. This quotient is the same in all nodes in an undirected graph (even for large- and low-degree nodes, that is, for hubs and non-hubs as well), but may differ significantly from node to node in directed graphs. We suggest to assign importance to non-hub nodes with large PageRank/in-degree quotient. Consequently, our method gives high scores to nodes with large PageRank, relative to their degrees: therefore non-hub important nodes can easily be identified in large networks. We demonstrate that these relatively high PageRank scores have biological relevance: the method correctly finds numerous already validated drug targets in distinct organisms (*Mycobacterium tuberculosis, Plasmodium falciparum and MRSA Staphylococcus aureus*), and consequently, it may suggest new possible protein targets as well. Additionally, our scoring method was not chosen arbitrarily: its value for all nodes of all undirected graphs is constant; therefore its high value captures importance in the directed edge structure of the graph.

## Introduction

Methods analyzing biological networks are gaining significant interest because of their availability in large public repositories [1]–[9]. Finding important nodes in these protein-protein interaction or metabolic networks may lead to the identification of novel drug targets. The FDA approved drugs target presently only 324 human and pathogen proteins [10] from at least tens of thousands of possible proteins, therefore any well-founded method that may help to identify new ones has a substantial value.

Selecting important nodes that would serve as drug targets is a difficult task. In the literature, important nodes frequently means nodes with high degree (i.e., with many connecting edges, leading to a great number of neighboring nodes); these nodes are called “hubs” and “superhubs” [11], [12]. The proteins, corresponding to these hubs are mainly catalyzing vital biochemical reactions in metabolic networks [13] or their neighbor-set are robust: they are hardly changed in biological processes [12].

Targeting hub proteins with numerous vital functions with inhibitors may lead to unwanted off-target effects [14], [15] in the living cell, since any interventions involving these hub proteins may effect a large number of other processes and proteins as well.

In the present study we restrict our attention to metabolic networks: here the nodes are biochemical reactions, and reactions *A* and *B* are connected with a directed edge (*A,B*) if a product of reaction *A* enters reaction *B* as a substrate or a co-factor. In a given organism reactions can be corresponded to enzymes, catalyzing them. This correspondence can be made easily by inspecting the underlying database: we applied the KEGG database [16] for this mapping.

In the analysis of metabolic networks, large or very large degree nodes (hubs or superhubs, corresponding to “currency metabolites” [17]) usually need special attention if we want to compensate for their overwhelming weight: these nodes are sometimes simply removed from the network in a pre-processing step [18], changing significantly the connectivity properties of the network. We do not remove the high-degree nodes in the networks, since then the whole graph would be changed significantly. We rather introduce a new scoring function, that compensates the important small degree nodes against hubs or superhubs.

## Results and Discussion

In the present work we introduce a method for finding relevant nodes (e.g., possible new protein targets) in networks with directed edges, especially in metabolic networks, that is robust and can compensate small degree nodes against large degree nodes, therefore our method does not need pre-processing steps to remove vertices, corresponding to “currency metabolites”. We also show that our method successfully identifies numerous already verified relevant protein targets, and therefore, may be used to identify novel ones in other directed networks as well.

Let us note that *proving* that several highly scored proteins in our method are new, still unknown protein targets, would require multi-year wet-lab work (i) for developing new inhibitors against the new, suggested protein targets; (ii)proving that the inhibitors have significant biological activity, (iii) proving that the inhibitors inhibit the new target protein, and not some other enzymes. That work is out of scope of the present theoretical paper. Therefore our proof contains references to target proteins, discovered earlier independently from us, that gained high scores in our method, solely by graph theoretic analysis of the underlying metabolic graphs.

We demonstrated in [19] that the PageRank of vertices [20], applied first in the Google web-search engine [20] for identifying important web pages, can also be used in the robust analysis of protein networks to identify important nodes. Here “robustness” means that changes in the less interesting parts of the network will not cause significant changes in the PageRank of the more important nodes (see [19] for a more exact statement).

It is known, however, that large degree nodes *usually* have large PageRank on the average [21], therefore PageRank alone cannot always compensate the overweight of hubs and superhubs in the identification of important nodes in a network.

Here we suggest to use for the scoring the importance of nodes in metabolic networks the “relativized personalized PageRank”. Let *G* be a directed graph. The PageRank [20] of graph *G* is the limit probability distribution of the random walk, defined by the column-stochastic transition matrix.
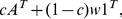
(1)where *A* is row-stochastic transition matrix, prepared by normalizing the rows of the adjacency matrix of graph *G* [22], 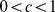 is the damping constant, **1** is the all-1 column-vector, and vector *w* with non-negative coordinates, satisfying 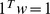, is the personalization vector. In the original, non-personalized version of the PageRank of an *n*-vertex graph, 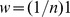. We use everywhere in this work the value 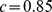.

We must note that the role of the personalization vector, *w*, was originally to capture the personal interests of the web-surfers to compute a personalized ranking of the web-pages for web-search engines [20]. If no personalization is given (when 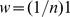) that means that in the teleporting step of the walk, each vertex can be visited with the same probability. Personal interests of the web-surfers can be introduced into the random walk by increasing the probability of web-sites interesting to the surfer by increasing their probability in the distribution given in vector *w*.

In [19] we have shown that if vector *w* is personalized to proteins, appearing in higher concentrations in proteomics analysis of certain diseases, then this personalized PageRank may emphasize other closely related proteins to the disease, that eventually did not appear in the proteomics analysis, either because of their low concentration or by their cellular compartmentalization.

It is *demonstrated* [23] through computational simulations, that in undirected graphs, the PageRank of a node is approximately proportional to the degree of that node; consequently, for undirected graphs, the PageRank will not yield additional information on node relevance, relative to degree.

In [22] we *proved* that in the case of undirected graphs, the PageRank of the vertices are *exactly proportional* to their degrees if and only if the coordinates of the personalization vector *w* are proportional to the degrees of the vertices, that is:
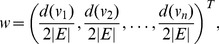
(2)where 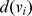 denotes the degree of vertex 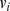, and 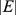 denotes the number of the edges in graph *G*.

In other words, the PageRank, defined by the limit probability distribution of (1) with *w* given in (2) is exactly *w* for undirected graphs. Therefore if we divide coordinate *i* by 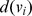, for 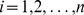, then we get the same constant for each coordinates.

This means that dividing the PageRank personalized by vector *w*, by the degrees, we factor out high- or low degreeness from the score: for every vertex the ratio is the same.

We would like to introduce a similar measure for directed graphs, that factors out the degrees in above sense, and the resulted scores would allow to reach high values for low-degree nodes, too.

More exactly, we define for the directed graph *G* the vector.
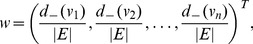
(3)where 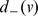 is the in-degree of vertex *y* (i.e., the number of directed edges pointing to vertex *v*).

Now we can define the “relativized personalized PageRank” of graphs as follows: Let PPageRank denote the PageRank given by the stationary distribution of the walk of equation (1) computed with *w* of equation (3), then.
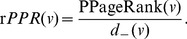
(4)

- Clearly, in *undirected graphs*, our relativized PageRank 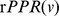 is exactly constant, *i.e.,* it is completely independent from the node (and its degree). Therefore, in undirected graphs, it is the same for large- and small degree nodes, so in *directed graphs* its high value may describe a sort of “intrinsic” importance of the node, independent from its high- or low degree, and depending only on the directed graph-connectivity structure of the network. We find this to be an important property, since it shows that our score function was not chosen “arbitrarily”, it “factors out” the undirected degree from the scoring.
- The PageRank of large degree nodes are *on the average,* large in any graph (see [21] for a much more exact statement). Dividing the PageRank of node *v* by its in-degree will compensate the small in-degree nodes, since their PageRank is divided only with a small number. Therefore the small in-degree, relevant nodes may stand out in this scoring function. This scoring function will usually not give high scores for network hubs, but these hubs can easily be identified by simple degree counting, and does not need more sophisticated tools.

As we demonstrate here in the application examples, the new scoring method will choose low degree nodes with proven biological interest. Therefore, the presented approach can effectively be used to find promising drug targets because the reactions (nodes) with high PageRank and low in-degree correspond to essential reactions.

### Application Examples in Microbial Networks

For demonstrating the applicability of this new scoring function, we present several examples from much researched pathogen microorganisms. We show that several well known protein targets correspond to highly scored nodes, and this fact may imply that other highly scored nodes may be promising, non-hub, new drug targets. We would like to stress that in identifying new possible drug targets we applied only the rPPR score of us (4), and have not used structural or functional annotations of the proteins, just their positions in the metabolic networks.

### Mycobacterium Tuberculosis

Our **first example** is the mycolic acid metabolic pathway [24] in the *Mycobacterium tuberculosis* bacterium. Since mycolic acid synthesis is missing in eukaryotes, targeting specific enzymes in this pathway seems to be a natural choice for target search.

**Figure 1:**
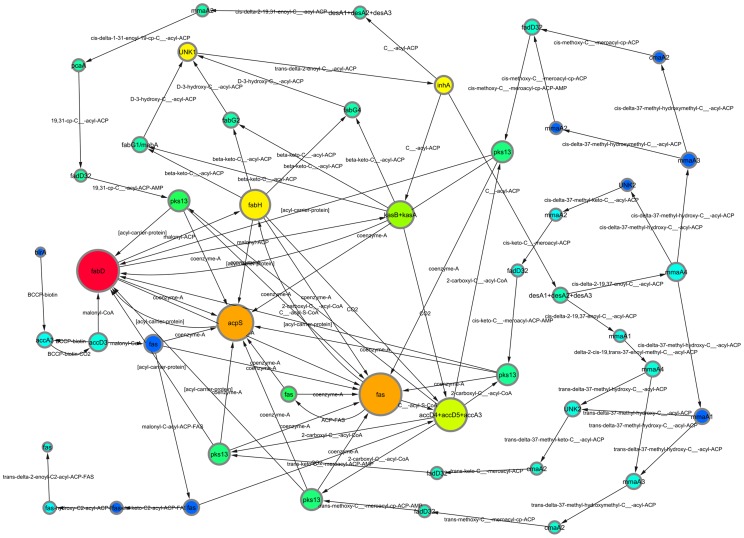
The network of the mycolic acid synthesis [24]. Node sizes correspond to the degree of the node, node color correspond to the personalized PageRank of the node: warmer colors mean larger PageRank. Note the small but yellow node labeled by InhA in the upper central part of the picture.

rPPR was computed for the mycolic acid pathway, the result is depicted on Figure 1. The size of the nodes are corresponding to the degree of the vertex, and the color of the node to the rPPR of the vertex: the warmer the color the higher the rPPR score.

The yellow inhA node in the upper right quadrant of Figure 1 has in-degree 1, therefore it is not a hub at all. On the other hand, by Table 1, its rPPR is far the highest in the network. This scoring correlates well with the fact that inhA (long-chain enoyl-acyl carrier protein reductase) is one of the oldest known and most important target of TB drugs isoniazid and ethionamide, and also the prime target of several novel drugs under development today [25]–[27].

**Table 1:** The list of six nodes with the rPPR scores in the mycolic acid pathway of the *Mycobacterium tuberculosis.*

*Node*	*PPR in*	*Degree*	*in-degree*	*PPR in / in-degree*
inhA	0.049	4	1	0.049
fabH	0.058	8	2	0.029
fas	0.029	3	1	0.029
kasB kasA	0.045	7	2	0.023
UNK1	0.055	4	3	0.018
fabD	0.133	12	8	0.017

The node with the second highest rPPR, labeled by FabH (see Table 1) is also a well-researched possible TB drug target [28]–[31].

Our **second example** concerns the whole metabolic network (not only the mycolic acid pathway) of the *Mycobacterium tuberculosis*. The network contains 947 nodes, the rPPR scores and the vertices with non-zero in-degrees are given in Table S1 in the on-line supporting material.

Table 2 shows the list of the nodes with the highest rPPR score. The highest and second highest scoring reactions correspond to the protein pdxH, a putative pyridoxine 5′-phosphate oxidase (Rv2607) is reported [32] having strongly different putative binding pocket than any other member of its enzyme family.

**Table 2:** The list of the 11 nodes in the metabolic network of the tuberculosis bacterium with the highest rPPR score.

*reaction ID*	*PPR in*	*Degree*	*in-degree*	*PPR in/in-degree*	*protein correspondence*
R00278	0.0061	3	2	0.0030	Rv2607 pdxH
R00277	0.0061	3	2	0.0030	Rv2607 pdxH
R01209	0.0025	7	1	0.0025	Rv0189c ilvD
R03051	0.0028	3	2	0.0014	Rv3001c ilvC
R06905	0.0013	1	1	0.0013	bnsG
R03968	0.0020	4	2	0.0010	Rv2987c(leuD) Rv2988c(leuC)
R04942	0.0020	3	2	0.0010	Rv1077 cysM
R04440	0.0020	4	2	0.0010	Rv3001c(ilvC)
R05071	0.0027	5	3	0.0009	Rv3001c(ilvC)
R01214	0.0046	12	6	0.0008	Rv2210c(ilvE)
R01215	0.0046	12	6	0.0008	Rv0337c(aspC)

The full table is available as Table S1 in the on-line supporting material.

Very recently it is reported [33] that the downregulation of the third largest scoring protein with gene name ilvD (Rv0189c, a dihydroxyacid dehydratase) affects the growth of *Mycobacterium tuberculosis in vitro* and in mice.

The sixth highest scoring hit, the leuD gene (Rv2987c) is shown to be essential in *Mycobacterium tuberculosis* even in macrophages [34].

The seventh highest-scoring protein is cysM (Cysteine synthase, Rv1077), is reported [35] to have intermediate protection properties and in sulfur donor selectivity, and also is known to play a main role in a mycobacteria-specific, alternative cysteine biosynthesis pathway [36].

The third, fourth, eighth, ninth and tenth highest scored hits are related to branched chain amino acid (BCCA, comprises leucine, isoleucine and valine) synthesis of the bacterium. Examples were shown in [37] that these proteins may serve as drug targets.

### Plasmodium Falciparum

The metabolic network for *Plasmodium falciparum* contains 450 nodes. Table 3 shows eleven of the highest rPPR scoring vertices, while the full table is available as Table S2 in the supporting on-line material.

**Table 3:** The list of the eleven nodes with the highest rPPR in *Plasmodium falciparum*.

*reaction ID*	*PPR in*	*Degree*	*in-degree*	*PPR in/in-degree*	*protein correspondence*
R00173	0.0123	3	2	0.0061	pyridoxal kinases
R00174	0.0123	3	2	0.0061	pyridoxal kinases
R03316	0.0043	8	2	0.0021	2-oxoglutarate dehydrogenase
R01890	0.0024	3	2	0.0012	cholinephosphate cytidylyltransferase
R01021	0.0024	3	2	0.0012	choline kinase
R07604	0.0020	8	2	0.0010	branch.-chain alpha keto-acid dehydr.
R07602	0.0020	8	2	0.0010	branch.-chain alpha keto-acid dehydr.
R07600	0.0020	8	2	0.0010	branch.-chain alpha keto-acid dehydr.
R01961	0.0018	4	2	0.0009	hexokinase
R01940	0.0008	3	1	0.0008	2-oxoglutarate dehydrogenase
R01626	0.0081	19	10	0.0008	PfMCAT

The full table with 450 nodes is available as Table S2 in the supporting on-line material.

Reactions of the highest and second highest score (R00174 and R00173, resp.) are corresponded to pyridoxal kinases (EC:2.7.1.35) that are shown to be targets or Roscovitine in [38] and a possible target in the malaria parasite in [39]. It is reported in [40] that inhibiting pyridoxal 5-phosphate-dependent enzymes kills the parasite efficiently.

The fourth highest scoring R01890, corresponding to PfCCT, cholinephosphate cytidylyltransferase, is shown to be the target of a potent experimental malaria drug, PG12 in [41].

The fifth highest scoring hit, R01021, corresponds to choline kinase, that is reported to be the target of hexadecyltrimethylammonium bromide in the malaria parasite in [42].

The sixth, seventh and eights highest scored reactions (R07604, R07602, R07602) are corresponded to branched-chain alpha keto-acid dehydrogenases, and they are shown to be specific in function in *Plasmodium falciparum*, therefore they may serve as a selective target [43].

In [44] it is shown that the ninth hit R01961, corresponding to hexokinase, can be viable target in *Plasmodium falciparum*.

The tenth highest scoring reaction is R01940 (EC:1.2.4.2, 2-oxoglutarate dehydrogenase (OGDH) E1 component). The related pathways include vitamin B metabolism, a recently suggested target [45], and relates to alpha-ketoacid dehydrogenase multienzyme complexes, with specificity for *Plasmodium falciparum*. Glutamate dehydrogenase, catalyzing a closely related reaction, was suggested also as new drug target recently [46].

With unusually high in- and outdegrees, the eleventh highest scoring reaction, R01626 is corresponded to enzyme PfMCAT, that is shown to be essential in fatty acid synthesis of the parasite in [47].

### MRSA Staphylococcus Aureus

We applied the metabolic data of the MRSA *Staphylococcus aureus* SAA strain (USA300_FPR3757 (CA-MRSA)) for the network generation. The network contains 803 network nodes. The data of all the nodes are available as Table S3 in the on-line supporting material, while nine nodes with the largest score is given on Table 4.

**Table 4:** The list of nine nodes with the highest rPPR score in MRSA *Staphylococcus aureus*.

*reaction ID*	*PPR in*	*Degree*	*in-degree*	*PPR in/in-degree*	*protein correspondence*
R00174	0.0083	3	2	0.0041	phosphomethylpyrimidine kinase (EC:2.7.4.7)
R00173	0.0083	3	2	0.0041	pyridoxal phosphate phosphatase
R07600	0.0047	13	2	0.0024	2-oxoisovalerate dehydrogenase
R02272	0.0045	4	2	0.0023	hemL
R04109	0.0039	3	2	0.0019	hemA
R03316	0.0032	13	2	0.0016	sucA
R00036	0.0027	4	2	0.0013	hemB
R07604	0.0026	13	2	0.0013	2-oxoisovalerate dehydrogenase
R01209	0.0013	8	1	0.0013	ilvD

The full table with 450 nodes is available as Table S3 in the supporting on-line material.

The two highest scoring reactions relate to vitamin B metabolism and reported to be important in SAA in [48].

R02272, R04109 and R00036 correspond to the hemL, hemA and hemB genes, respectively, of the heme synthesis. Inactivation of the hemB gene leads to an aberrant form of the bacterium, the small colony variant (SCV) [49]. Most recently, [50] also reports the significance of these genes.

R07600, R07604 and R01209 (ilvD) play a main role in branched-chain amino acids biosynthesis pathway of the bacterium [51].

## Materials and Methods

The mycolic acid network was prepared using the pathways published in [24]. The nodes are labeled by the gene names of the enzymes, and two nodes *X* and *Y*, corresponding to enzymes denoted by their gene names, are connected by a directed edge from *X* to *Y* if and only if there exists a substrate *u*, leaving the reaction, catalyzed by enzyme *X*, that enters the reaction, catalyzed by enzyme *Y*. Substrate *u* labels the directed edge from *X* to *Y*.

For creating Figure 1, we applied Cytoscape [52] for the data published in [24]. A high resolution version of Figure 1 is available as Figure S1 in the on-line supporting material.

The metabolic networks for *Mycobacterium tuberculosis, Plasmodium falciparum* and *MRSA Staphylococcus aureus* were generated from the KEGG database [16], data downloaded on December 13, 2010. The network nodes were labeled by the KEGG reaction ID's. The full datasets, containing the degrees and the PageRanks of the nodes are available as on-line supporting material.

PageRank was computed using the NetrworkX Python library [53] (downloadable from the Los Alamos national Laboratory http://networkx.lanl.gov/) with our Python script ppr_pub.py, downloadable from http://uratim.com/rPPR.

### Conclusions

Traditionally, the discovery of novel protein targets relies on multi-decade long work on several biochemical reactions in living organisms. New tools and insights make possible that the systems biology would also suggest new possible targets, by examining the protein-protein or protein-metabolite interactions of the cell. We believe that using well-developed methods from graph theory and computer science will yield significant results in biology. In particular, ordinary PageRank can help to evaluate important nodes and pathways in directed networks, especially when relativized with other network properties, like the in-degree of nodes. We think that the present method is capable for identifying low-degree nodes with high intrinsic metabolic functionality in networks, clearly and automatically.

The rPPR measure introduced in Equation (4) has the following remarkable property: its value is the same for each vertex (either with large or small degrees) of an undirected graph, while for directed graphs, rPPR may change significantly from vertex to vertex, and it captures importance due to the directions of the edges in the graph.

Our method gives high scores to nodes that have high PageRank relative to their degrees, therefore clearly and easily identifies important nodes of low-degrees in biological networks. Consequently, the method and the scoring function can be effectively used to find promising drug targets in metabolic networks, because the reactions (nodes) with high PageRank and low in-degree correspond to essential reactions.

## Supporting Information

Table S1 **The degree, in-degree, PPR and rPPR data for the metabolic network of** ***Mycobacterium tuberculosis*** **.**
(XLS)

Table S2 **The degree, in-degree, PPR and rPPR data for the metabolic network of of** ***Plasmodium falciparum*** **.**
(XLS)

Table S3 **The degree, in-degree, PPR and rPPR data for the metabolic network of MRSA bacterium.**
(XLS)
